# Generalizability of digital speech and language assessments in MCI/mild AD across four non‐English languages

**DOI:** 10.1002/alz70861_107986

**Published:** 2025-12-23

**Authors:** Martina De Lillo, Mengdan Xu, Michael J. Spilka, Francesca K Cormack, Jessica Robin, Katharina Fuchs, Christine Wenzkowski, Michael Schaeffer

**Affiliations:** ^1^ Cambridge Cognition, Cambridge UK; ^2^ Cambridge Cognition, Toronto, ON Canada; ^3^ Vivoryon Therapeutics NV, Halle Germany

## Abstract

**Background:**

Neurodegenerative diseases affect speech production. Previous studies show various acoustic and linguistic changes in Mild Cognitive Impairment (MCI) and Alzheimer's disease (AD) speech patterns, such as more pauses and lower usage of nouns. However, most research has focused on English speakers. This analysis examines speech features in MCI and AD patients across four languages to determine if digital speech assessments yield comparable results across different languages.

**Method:**

This study included 244 patients with MCI or mild AD enrolled in the Phase 2b VIVIAD trial of varoglutamstat, in four language groups: Danish (*n* =84), German (*n* =59), Dutch (*n* =40), and Spanish (*n* =61). A picture description task was administered to all patients at baseline using instructions in their respective languages. Five speech features including number of picture objects described, number of nouns, speech rate, pronoun‐to‐noun ratio and average of word length were chosen based on previous analyses of speech patterns in MCI/AD, and a composite score was calculated combining the five measures. We tested the interrelationships among the features in each language and examined whether the speech features were related to another measure of cognitive function, the Mini‐Mental State Exam (MMSE). Associations were examined using a correlational approach.

**Result:**

There was variation in the magnitudes of the correlations between speech features in the different languages, but the directions of the associations were generally consistent across the four languages in the study. In all four languages, significant correlations with the MMSE were found with both the composite score (‐0.46 ≤ *r* ≤ ‐0.26) and the object content score (0.27 ≤ *r* ≤ 0.42).

**Conclusion:**

Together, these results indicate that there are consistent interrelationships among different speech features across languages. In addition, in the four languages examined, the speech composite score and the number of objects correctly described related to a standard measure of cognition, the MMSE. These results suggest that digital measures of speech and language in MCI/AD are generalizable across languages and relate to cognitive ability more generally. In addition, a composite score of multiple speech features may be more sensitive to cognitive impairment than individual speech metrics.

